# Seasonal malaria chemoprevention in an area of extended seasonal transmission in Ashanti, Ghana: an individually randomised clinical trial

**DOI:** 10.1111/tmi.12642

**Published:** 2015-12-16

**Authors:** Harry Tagbor, Gifty Dufie Antwi, Princess Ruhama Acheampong, Constance Bart Plange, Daniel Chandramohan, Matthew Cairns

**Affiliations:** ^1^ School of Public HealthKwame Nkrumah University of Science and TechnologyKumasiGhana; ^2^ Centre for Global Health ResearchJuabenGhana; ^3^ National Malaria Control ProgrammeGhana Health ServiceAccraGhana; ^4^ Department of Disease ControlLondon School of Hygiene and Tropical MedicineLondonUK; ^5^ MRC Tropical Epidemiology GroupLondon School of hygiene and tropical medicineLondonUK

**Keywords:** seasonal malaria chemoprevention, community case management, artemisinin‐based combination therapies, individually randomised, placebo‐controlled trial, Ashanti, Ghana, chimioprévention saisonnier du paludisme, malaria, prise en charge communautaire des cas, thérapies de combinaison à base d'artémisinine, randomisé individuellement, essai contrôlé par placebo, Ashanti, Ghana

## Abstract

**Objective:**

To investigate the effectiveness of seasonal malaria chemoprevention (SMC) and community case management with long‐acting artemisinin‐based combination therapies (ACTs) for the control of malaria in areas of extended seasonal malaria transmission.

**Method:**

Individually randomised, placebo‐controlled trial in the Ashanti Region of Ghana. A total of 2400 children aged 3–59 months received either: (i) a short‐acting ACT for case management of malaria (artemether‐lumefantrine, AL) plus placebo SMC, or (ii) a long‐acting ACT (dihydroartemisinin‐piperaquine, DP) for case management plus placebo SMC or (iii) AL for case management plus active SMC with sulphadoxine‐pyrimethamine and amodiaquine. SMC or placebo was delivered on five occasions during the rainy season. Malaria cases were managed by community health workers, who used rapid diagnostic tests to confirm infection prior to treatment.

**Results:**

The incidence of malaria was lower in children given SMC during the rainy season. Compared to those given placebo SMC and AL for case management, the adjusted hazard ratio (aHR) was 0.62 (95% CI: 0.41, 0.93), *P* = 0.020 by intention to treat and 0.53 (95% CI: 0.29, 0.95), *P* = 0.033 among children given five SMC courses. There were no major differences between groups given different ACTs for case management (aHR DP *vs*. AL 1.18 (95% CI 0.83, 1.67), *P* = 0.356).

**Conclusion:**

SMC may have an important public health impact in areas with a longer transmission season, but further optimisation of SMC schedules is needed to maximise its impact in such settings.

## Abstract (fr)

**Objectif:**

Etudier l'efficacité de la chimioprévention saisonnière (CPS) contre le paludisme et de la prise en charge communautaire des cas avec des thérapies de combinaison à base d'artémisinine (TCA) à action prolongée pour la lutte contre le paludisme dans les zones de transmission saisonnière prolongée.

**Méthode:**

Essai contrôlé par placebo, individuellement randomisé, dans la région Ashanti, au Ghana. 2400 enfants âgés de 3 à 59 mois ont reçu soit: (1) une TCA de courte durée d'action (artéméther‐luméfantrine, AL) pour la prise en charge des cas de paludisme, plus une CPS placebo, ou (2) une TCA de longue durée d'action (dihydroartémisinine‐pipéraquine, DP) pour la prise en charge des cas, plus une CPS placebo ou (3) AL pour la prise en charge des cas, plus une CPS active (sulfadoxine‐pyriméthamine et amodiaquine). La CPS ou le placebo ont été administrés à cinq reprises au cours de la saison des pluies. Les cas de paludisme ont été pris en charge par des agents de santé communautaires, qui ont utilisé des tests de diagnostic rapide pour confirmer l'infection avant le traitement.

**Résultats:**

L'incidence du paludisme était plus faible chez les enfants qui ont reçu la CPS pendant la saison des pluies. Par rapport à ceux ayant reçu la CPS placebo et AL pour la prise en charge des cas, le rapport de risque ajusté (aHR) était de 0,62 (IC95%: 0,41 à 0,93), P = 0,020, en intention de traiter et de 0,53 (IC95%: 0,29 à 0,95), P = 0,033, chez les enfants qui ont reçu cinq doses de CPS. Il n'y avait pas de différences majeures entre les groupes ayant reçu différentes TCA pour la prise en charge des cas (aHR pour DP vs AL : 1,18 (IC95%: 0,83 à 1,67), P = 0,356).

**Conclusion:**

La CPS peut avoir un impact important sur la santé publique dans les régions avec une saison de transmission plus longue, mais une optimisation supplémentaire des périodes de CPS est nécessaire afin de maximiser son impact dans de tels contextes.

## Abstract (es)

**Objetivo:**

Investigar la efectividad de la quimioprevención estacional de la malaria (QEM) y el manejo comunitario de casos con una combinación de fármacos basada en la artemisinina (ACTs) para el control de la malaria en áreas de transmisión estacional de larga duración.

**Método:**

Ensayo aleatorizado de forma individual, controlado con placebo, en la región de Ashanti en Ghana. 2400 niños con edades entre los 3‐59 meses recibieron una de las siguientes: (1) ACT de corta duración para el manejo del caso de malaria (artemeter‐lumefantrina, AL) más QEM placebo, o (2) ACT de larga duración (dihidroartemisinina‐piperaquina, DP) para el manejo del caso más QEM placebo o (3) AL para el manejo de caso más QEM activo con sulfadoxina‐pirimetamina y amodiaquina. Se entregaron el QEM o placebo en cinco ocasiones durante la estación de lluvias. Los casos de malaria los manejaron trabajadores sanitarios comunitarios, que utilizaron pruebas de diagnóstico rápido para confirmar la infección antes de administrar el tratamiento.

**Resultados:**

La incidencia de malaria era menor entre niños que recibieron QEM durante la estación de lluvias. Comparados con aquellos que recibieron QEM placebo y AL para el manejo de casos, el cociente de riesgo ajustado (aHR) era 0.62 (IC 95%: 0.41, 0.93), P = 0.020 en intención de tratar y 0.53 (IC 95%: 0.29, 0.95), P = 0.033 entre niños que recibieron cinco dosis de QEM. No había grandes diferencias entre los diferentes grupos de ACTs para el manejo de casos (aHR DP vs. AL 1.18 (IC 95% 0.83, 1.67), P = 0.356).

**Conclusión:**

La QEM podría tener un impacto importante en la salud pública de áreas con una estación de transmisión más larga, pero ser requiere optimizar la programación de QEM para maximizar su impacto en estos lugares.

## Introduction

Seasonal malaria chemoprevention (SMC) is a highly effective means to control malaria in areas with a short rainy season, where a large proportion of the annual malaria burden occurs within a few months of the year 1. SMC involves monthly administration of the long‐acting antimalarial drugs sulfadoxine‐pyrimethamine and amodiaquine (SP‐AQ) to all children under five years of age, regardless of whether or not they are infected, and is a policy recommended by WHO for malaria control in the Sahel and sub‐Sahel regions of Africa 2. To date, most studies of SMC have taken place in areas with a short rainy season 3, 4, 5, 6, 7, and current evidence suggests that, for SMC to be appropriate, 60% of the total annual burden of malaria should occur within 4 months of the year 8. However, the basic approach used in SMC (monthly ‘cycles’ of SP‐AQ during the period of highest risk) could be adapted to cover a longer period of risk in areas where malaria is transmitted for longer each year, and the burden is spread more evenly over a longer period. There are large highly populated areas of West Africa where SP and AQ are still highly effective, and which have rainy seasons ranging from 5 to 6 months in length 8, 9. If SMC were effective in such areas, this would substantially increase the overall population that could be protected by SMC and would help to reduce the large burden of malaria in children in West Africa. To date, only two other studies have considered SMC given over a longer period: a study in Hohoe, Ghana, where artesunate‐amodiaquine was given monthly over 6 months 10, and a study in Senegal, where SP‐AQ was administered monthly for 5 months (clinicaltrials.gov identifier: NCT01449045). These trials have not resulted in changes in practice because the former used an artemisinin‐based combination for SMC, whereas the latter occurred in an area where 4 months of SMC would likely have been sufficient 11.

A second form of drug‐based prevention that would be easier for resource limited countries to implement would be to treat malaria patients with long‐acting artemisinin combination therapies (LACTs), such as dihydroartemisinin‐piperaquine (DP) or artesunate‐mefloquine (AS‐MQ), which protect children against further malaria episodes for several weeks 12, 13. The period of ‘post‐treatment prophylaxis’ after use of LACTs will be more useful in certain epidemiological situations than in others, for example in high burden areas where re‐infection is likely soon after treatment, and particularly in high burden seasonal settings 14, 15. In such areas, LACTs could be used in combination with SMC, or instead of SMC where implementation is not feasible, for example where the malaria transmission season extends over a larger part of the year. Choice of first‐line antimalarial combination is not usually made with consideration of malaria epidemiology, but this could be rationalised if there was better evidence to show where it would be effective.

To investigate these issues, we undertook a trial in the Ashanti region of Ghana to estimate the protection provided by SMC administered over 5 months and by a long‐acting ACT used for malaria case management. A previous study in this area showed that amodiaquine‐artesunate administered every 2 months reduced fevers presumptively treated as malaria by community‐based health workers (CHWs) by 37% (by intention to treat), and by 61.5% in children who received all three courses over the malaria transmission season 16. However, the previous study was unable to confirm all fevers as malaria. CHWs resident in the study communities have since been trained to use rapid diagnostic tests to confirm malaria in children seeking treatment for illness following a standard algorithm and provide treatment for malaria using artemisinin‐based combination therapies.

## Methods

### Study population and screening

**Figure 1:**
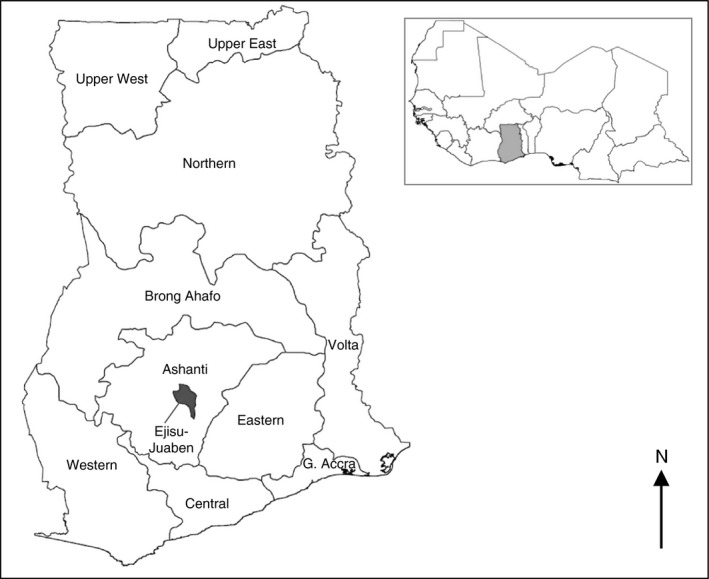
Location of the Ejisu‐Juaben District within the Ashanti Region of Ghana.

The study, registered with ClinicalTrials.gov [NCT01651416], was conducted among children aged 3–59 months living in 13 communities in the Kwaso subdistrict, Ejisu‐Juaben municipality, in the Ashanti Region of Ghana (Figure 1). The rainy season begins in May and lasts until October. Malaria transmission lags around 1 month behind, with transmission usually beginning to peak in June–July, and lasting until November. Community sensitisation and meetings with community leaders took place in early 2012 prior to the start of study activities. A census was carried out in March 2012 to enumerate the children under 5 years of age living in the area. Children were screened in July 2012, and if eligible enrolled and followed up for 12 months. Inclusion criteria were that the child was aged 3–59 months; was a permanent resident of one of the study communities and was able to take and retain oral medication. Exclusion criteria were any acute or chronic illness that needed further medical attention and any history of serious adverse reactions to drugs used in the study. As in areas where SMC has now been implemented, if a child was aged between 3 and 59 months at the time of enrolment, they could continue to receive SMC that year even if subsequently older than 59 months.

### Randomisation and allocation concealment

ID numbers comprising a four digit code and a check digit were randomly allocated to one of three intervention groups in a 1:1:1 ratio in permuted blocks of 12 using Stata version 13 (StataCorp, College Station, TX, USA). Treatment allocation was held in opaque sealed envelopes labelled only with the study ID number on the front. Upon enrolment to the study, the next envelope in the sequence was opened by the study team member to determine the treatment allocation of the child to be used throughout the study. This was carried out separately from the screening process; individuals screening children for eligibility were unaware of subsequent assignment. The three randomisation groups were (i) placebo SMC, plus a short‐acting ACT (artemether‐lumefantrine) for treatment of malaria episodes (hereafter the ‘AL group’), (ii) placebo SMC plus a long‐acting ACT (dihydroartemisinin‐piperaquine) for treatment of malaria episodes (the ‘DP group’) and (iii) active SMC with sulfadoxine‐pyrimethamine plus amodiaquine, plus artemether‐lumefantrine for treatment of malaria episodes (the ‘SMC group’).

### Sample size

Incidence of malaria in the region between 16 and 24 months of age was estimated to be 1.4 per child‐year by active case detection 17; we assumed passive case detection via community‐based health workers would detect 0.7 episodes per child‐year. A study including approximately 800 children in each group, allowing for a 10% loss to follow‐up, would have 90% power to detect a reduction of 20% in the malaria incidence rate between children given a long‐acting ACT and a short‐acting ACT. We assumed that SMC would reduce malaria incidence by 60% during the peak in transmission and by 40% over the study period. A study with 800 children in each group would have greater than 90% power to detect a difference of 25% between children given a long‐acting ACT and those given SMC, and very high power to detect larger differences between the SMC group and the short‐acting ACT.

### Blinding

To facilitate community‐based treatment of malaria with the assigned regimen (artemether‐lumefantrine or dihydroartemisinin‐piperaquine), and to ensure that children received the correct regimen if they attended at health centres in the study area, ID cards were colour‐coded according to intervention group and labelled with the regimen to be used for case management. The study was therefore open‐label with respect to the regimen used for case management but blinded with respect to whether seasonal malaria chemoprevention was active or placebo (members of the research team from KNUST/CGHR and LSHTM were aware of the allocation, but those who administered the SMC, and mothers/children were blinded).

### Follow‐up and interventions

The primary outcome was incidence of clinical malaria detected passively by community health workers, and at health centres serving the study area. If children presented with fever, history of fever or other symptoms suggestive of malaria, no other obvious causes of fever, no danger signs and tested positive for malaria by rapid diagnostic test (RDT, First Response *P. falciparum* HRP2/pLDH RDT (Premier Medical Corporation Ltd., Mumbai, India)), they received a full treatment course of either artemether‐lumefantrine (AL, Lumether, Kinapharma, Ghana) or dihydroartemisinin‐piperaquine (DP, Duo‐Cotexcin, Holley Pharmaceuticals, China) according to their intervention group. Children without symptoms of malaria, or those who tested negative by RDT, were referred to health centres or the hospitals serving the area. CHWs and health centres serving the study communities were supplied with RDTs and stocks of both ACTs; research staff stationed at health centres recorded illness visits made by study children to the outpatient clinics.

Between July and November 2012, study children received SMC with sulphadoxine‐pyrimethamine plus amodiaquine or identical placebos (Kinapharma, Ghana) on five occasions (once per month). SP/SP placebo and the first dose of AQ/AQ placebo were given under observation, and the two remaining AQ/AQ placebo tablets were administered by caregivers at home. Two age‐based dosages were used: children aged 1–4 years received 500/25 mg S/P and 153 mg AQ; infants received half this dose (250 mg/12.5 mg S/P, 76.5 mg AQ). In the days prior to SMC delivery, community health workers and field supervisors reminded mothers/caregivers that an SMC administration was due to take place; announcements were also made by local FM radio and by town criers. SMC or placebo was delivered by trained members of the study team at a central point in each community. For the final two cycles, community health workers followed up children who did not receive the SMC from the central point to administer the medicine. Children who reported illness or fever at the time of SMC were screened with a rapid diagnostic test, and if positive treated with an ACT according to their intervention group. Caregivers were followed up by CHWs between 4 and 7 days after each SMC administration to ask about adverse events and adherence to the SMC regimen using a standard form.

### Cross‐sectional surveys

At enrolment, information was collected on basic demographic characteristics, household facilities (drinking water source, toilet facilities, cooking fuel, roof construction and floor material), durable household assets and ownership, age, condition and use of insecticide‐treated nets. Blood samples were taken for determination of haemoglobin and malaria parasitaemia. Haemoglobin was measured using 301 Hemocue^™^ analysers (HemoCue, Angelholm, Sweden). Blood slides were prepared by trained staff and double read at the laboratory of the Centre for Global Health Research, Juaben, Ghana. Children were also asked about any current symptoms of illness using a structured questionnaire.

Two further cross‐sectional surveys were undertaken after the period when SMC was administered (January 2013), and at the end of the study period (July 2013). Blood samples were taken for determination of haemoglobin and malaria parasitaemia, and children were asked about any current illness symptoms.

### Data management and statistical methods

All data were double entered and any discrepancies verified. Consistency and range checks were also undertaken prior to locking the database for analysis, and linking records with the randomisation code. All analyses were undertaken in Stata version 13 (College Station, TX, USA). *A priori*, we expected that the malaria incidence would be highest in the AL group and lowest in the group given SMC, with the group given DP for malaria case management intermediate between these two (due to some children benefiting from the longer period of post‐treatment prophylaxis after malaria episodes). The AL group was therefore pre‐specified as the reference group for analyses. For the primary endpoint, the hazard ratio for multiple episodes of malaria was estimated by Cox regression models. Robust standard errors (i.e. the Andersen‐Gill extension of the Cox model) were used to account for clustering of failures within individuals; this estimates the total effect of the intervention which is of primary public health importance 18. The Efron method was used for tied failure times. Poisson regression with robust standard errors was used to obtain the prevalence ratio of *Plasmodium falciparum* parasitaemia and anaemia at the cross‐sectional surveys 19. Linear regression models were used to compare mean haemoglobin concentration. Covariates pre‐specified for inclusion in the analyses were age group, sex, socio‐economic status (SES), community of residence and use of an insecticide‐treated net (ITN).

### Ethical approval

The study was approved by the ethics committees of the London School of Hygiene and Tropical Medicine, UK, the University of Leeds, UK and the Kwame Nkrumah University of Science and Technology, Kumasi, Ghana.

## Results

**Figure 2:**
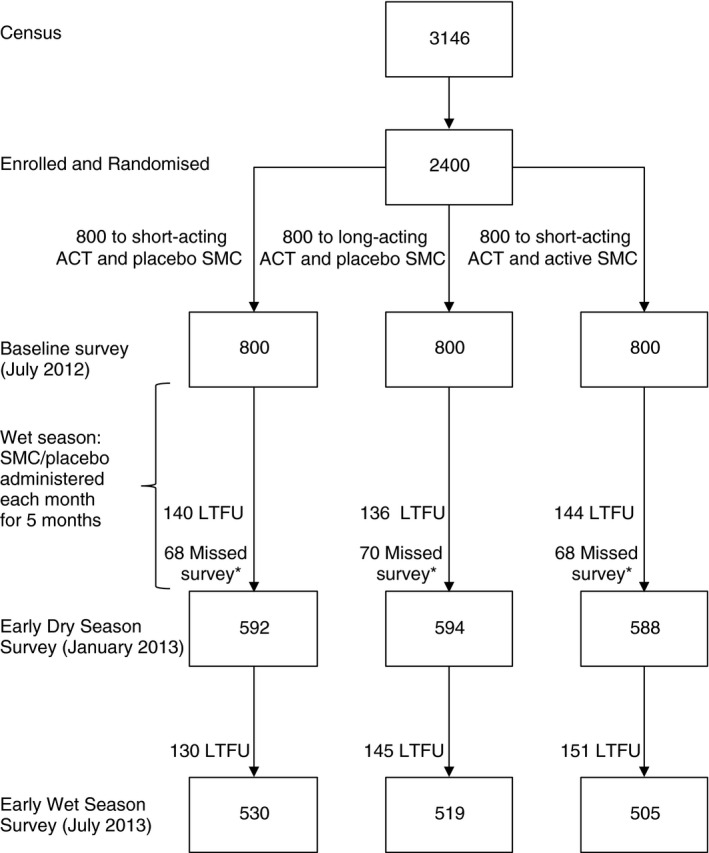
CONSORT diagram.

In July 2012, 2400 children were enrolled into the study (Figure 2). A total of 23% were infants, and 51.3% were male (Table 1). A community distribution of insecticide‐treated nets had occurred within the 6 months preceding the baseline survey; most nets were therefore new and insecticide treated, and 82.3% of children reported sleeping under an insecticide‐treated net the night before the survey. Despite the large number of children randomised, there were some imbalances between treatment groups at baseline. The AL only group had fewer infants than other groups (163, 20.4%); the SMC group had the largest proportion of infants (203, 25.4%) and proportionally fewer three‐year‐old children. Fewer SMC recipients used ITN: 78.8% *vs*. 84.3% and 83.9% in the AL and DP groups, respectively. Finally, there were some imbalances between groups in the number of children recruited from small communities (Table S1).

**Table 1:** Baseline characteristics of study children by study group

Characteristic		AL group		DP group		SMC group	
		No.	%	No.	%	No.	%
Age	0	163	20.4	184	23.0	203	25.4
	1	217	27.1	222	27.8	208	26.0
	2	157	19.6	128	16.0	149	18.6
	3	149	18.6	152	19.0	127	15.9
	4	114	14.2	114	14.2	113	14.1
Sex	Female	382	47.8	402	50.2	386	48.3
	Male	418	52.3	398	49.8	414	51.7
SES	Lowest	150	20.2	148	19.7	151	20.2
	Low	151	20.3	146	19.4	153	20.5
	Average	158	21.2	160	21.3	148	19.8
	High	145	19.5	152	20.2	140	18.7
	Highest	140	18.8	146	19.4	156	20.9
	Missing	56		48		52	
Owns a net	No	55	7.3	56	7.3	64	8.4
	Yes	702	92.7	710	92.7	696	91.6
	Missing	43		34		40	
Net last night	No	110	15.7	113	16.0	143	20.3
	Yes	592	84.3	594	84.0	563	79.7
	Missing	98		93		94	
ITN last night	No	110	15.7	114	16.1	150	21.2
	Yes	592	84.3	593	83.9	556	78.8
	Missing	98		93		94	
Malaria parasitaemia	No	569	71.8	583	73.7	587	74.6
	Yes	223	28.2	208	26.3	200	25.4
	Missing	8		9		13	
	Geometric mean parasite density	952.8	(756.0, 1200.9)	972.4	(746.8, 1266.2)	930.1	(718.9, 1203.2)
Hb	<5 g/dl	0	0	1	0.1	0	0
	5–7.99 g/dl	34	4.5	38	4.9	41	5.4
	8–10.99 g/dl	368	48.5	343	44.7	329	43.3
	≥11 g/dl	357	47	386	50.3	390	51.3
	Missing	41		32		40	
	Mean Hb (95% CI)	10.8	(10.7, 10.9)	10.9	(10.8,11.0)	10.9	(10.8, 11.0)

Malaria prevalence at baseline was 26.6% (95% CI 24.9%, 28.4%) and ranged from 13.3% to 57.6% in different communities, with infection generally more common in smaller communities (Figure S1). At baseline, 50.5% of children were anaemic (Hb < 11 g/dl), although most of this was mild, with only 4.98% of children having Hb <8 g/dl. One child had Hb <5 g/dl. The distribution of measured Hb was similar between the three groups (Figure S2).

The lowest coverage of SMC occurred at the third cycle of SMC, which was delivered by the study team at a central point in each community (Table 2). Qualitative research, to be reported separately, indicated that recent efforts to promote diagnosis prior to treatment of malaria in these communities may have reduced the perceived importance of obtaining drugs for prevention in healthy children. Access to the location where drugs were provided was identified as another important barrier, even though the distances within each community to the delivery point were small. In the fourth and fifth cycles, children who did not attend were followed up at home by community health workers, achieving higher coverage. Overall around 40% of children received all five cycles of SMC. Very few mothers reported adverse events to community health workers over the study period, although it is likely that the capture of this is not complete. Reported adherence to the three day course of SMC was very high: close to 100% in all communities and exactly 100% in a number of communities. However, it is unlikely that adherence was in reality this high, as during the qualitative component of this study, some caregivers were found to have tablets remaining that had not been administered.

**Table 2:** Coverage of SMC/Placebo SMC courses administered, by study group

	AL group		DP group		SMC group	
	No.	%	No.	%	No.	%
Coverage at each SMC round						
Round 1	800	100	800	100	800	100
2	565	70.6	581	72.6	555	69.4
3	464	58.0	478	59.8	468	58.5
4	548	68.5	574	71.8	535	66.9
5	662	82.8	676	84.5	670	83.8
Number of SMC rounds received						
1	54	6.8	44	5.5	50	6.3
2	96	12.0	80	10.0	82	10.3
3	133	16.6	131	16.4	149	18.6
4	191	23.9	213	26.6	228	28.5
5	326	40.8	332	41.5	291	36.4
At least 1 round	800	100	800	100	800	100
At least 2 rounds	746	93.3	756	94.5	750	93.8
At least 3 rounds	650	81.3	676	84.5	668	83.5
At least 4 rounds	517	64.6	545	68.1	519	64.9
5 rounds	326	40.8	332	41.5	291	36.4

### Cross‐sectional surveys

At the end of the rainy season, prevalence of malaria parasitaemia was lower in the SMC group than the AL group, 12.4% *vs*. 20.3%: prevalence ratio 0.61 (95% CI: 0.47, 0.80), *P* < 0.001. Prevalence was similar in the DP and AL groups, 19.5% *vs*. 20.3%: PR 0.96 (95% CI: 0.76, 1.21), *P* = 0.727 (Table 3). Prevalence of parasitaemia with density ≥3000 per μl (prevalence ≥5000 per μl in parentheses) was similar in all three groups, 4.3% (3.4%), 4.2% (3.6%) and 4.7% (3.4%) in the SMC, AL and DP groups, respectively. The distribution of haemoglobin was very similar in all intervention groups (Figure S2), and there were no differences in the prevalence of anaemia. At the end of the study in July 2013, there were no differences in prevalence of parasitaemia (23.2%, 22.0%, 20.5% in the SMC, AL and DP groups, respectively), or in the prevalence of anaemia (Table 3). Illness symptoms were similar between the study groups at both cross‐sectional surveys (Tables S6 and S7).

**Table 3:** Results of cross‐sectional surveys for malaria parasitaemia and haemoglobin

	January 2013						July 2013					
	AL group		DP group		SMC group		AL group		DP group		SMC group	
Malaria parasitaemia												
Uninfected (*N*, %)	470	79.7	478	80.5	514	87.6	411	78.0	411	79.5	384	76.8
*P. falciparum* (*N*, %)	120	20.3	116	19.5	73	12.4	116	22.0	106	20.5	116	23.2
Missing (*N*)	2		0		1		3		2		5	
Prevalence ratio (95% CI), *P*‐value	Reference		0.96 (0.76, 1.21), *P* = 0.727		0.61 (0.47, 0.80), *P* < 0.001		Reference		0.93 (0.74, 1.18), *P* = 0.552		1.05 (0.84, 1.32), *P* = 0.649	
*P. falciparum* ≥3000/μl (*N*, %)	25	4.2	28	4.7	25	4.3	39	7.4	41	8.0	50	10.0
Prevalence ratio (95% CI)			1.11 (0.66, 1.88), *P* = 0.692		1.01 (0.58, 1.73), *P* = 0.985				1.07 (0.70, 1.64), *P* = 0.741		1.35 (0.91, 2.02), *P* = 0.138	
Geometric mean density (95% CI)	961.8 (684.7, 1351.1)		1007.4 (727.6, 1394.9)		1500.8 (922.0, 2442.9)		1516.8 (1034.1, 2224.8)		1706.5 (1190.8, 2445.6)		1989.9 (1411.2, 2805.7)	
Haemoglobin												
Hb, g/dl (95% CI)	11.1 (11.0, 11.2)		11.1 (11.0, 11.2)		11.2 (11.1, 11.3)		11.0 (10.9, 11.2)		11.3 (11.1, 11.4)		11.1 (11.0, 11.2)	
5–7.99 g/dl (*N*, %)	20	3.4	18	3.0	16	2.7	25	4.8	15	2.9	19	3.8
8–10.99 g/dl (*N*, %)	248	42.2	245	41.3	240	41.1	220	42.0	189	36.6	217	43.3
≥11 g/dl (*N*, %)	319	54.3	330	55.6	328	56.2	279	53.2	313	60.5	265	52.9
Missing (*N*)	5		1		4		6		2		4	
Prevalence ratio vHb < 11 g/dl (95% CI), *P*‐value			0.97 (0.85, 1.11), *P* = 0.70		0.92 (0.80, 1.05), *P* = 0.218				0.83 (0.71, 0.96), *P* = 0.014		0.98 (0.85, 1.12), *P* = 0.754	
Prevalence ratio Hb < 8 g/dl (95% CI), *P*‐value			0.83 (0.43, 1.61), *P* = 0.586		0.85 (0.44, 1.63), *P* = 0.618				0.62 (0.32, 1.19), *P* = 0.147		0.77 (0.42, 1.43), *P* = 0.412	

### Incidence of malaria

**Figure 3:**
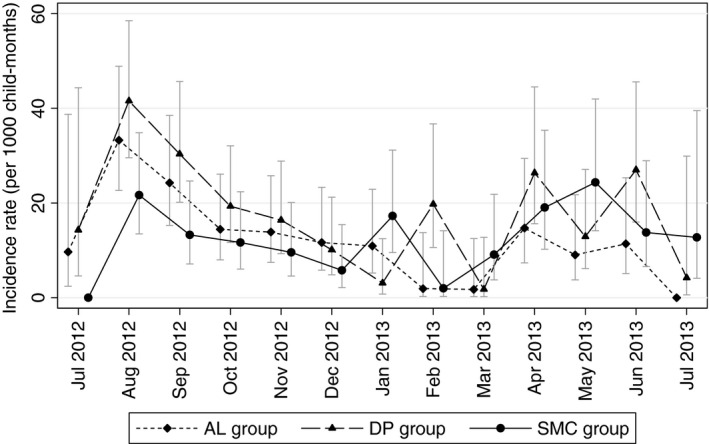
Monthly incidence of malaria by arm over the study period. Monthly incidence of malaria by study group. Error bars show 95% confidence intervals.

**Figure 4:**
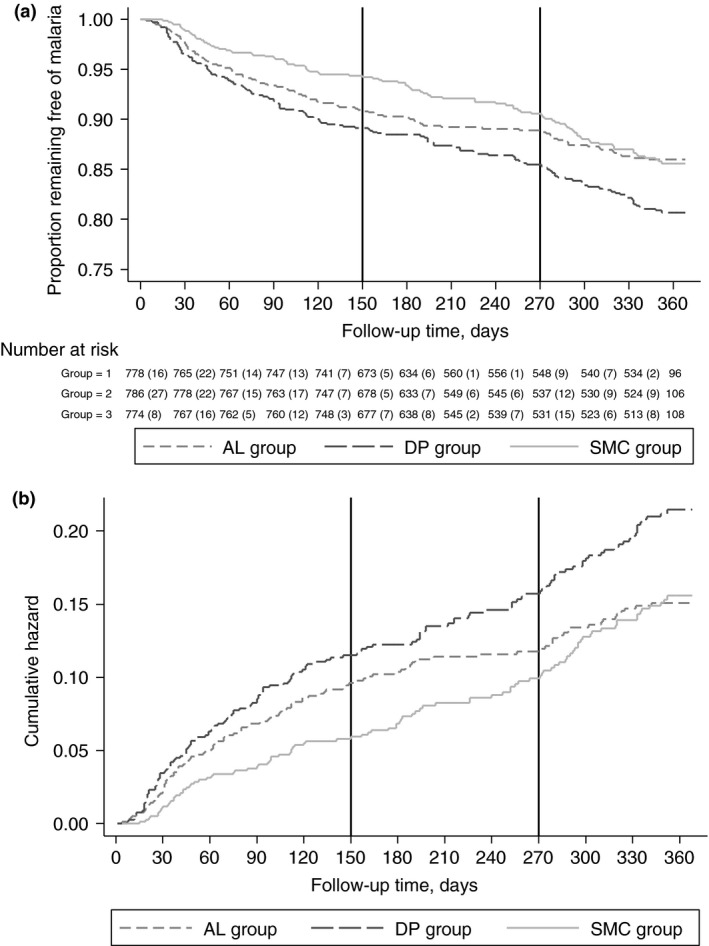
Kaplan–Meier and Nelson–Aalen plots of malaria incidence over the study period. (a) Kaplan–Meier estimate of the proportion of children remaining free of malaria over the course of the study period. (b) Nelson–Aalen estimate of the cumulative Hazard over the study period. Vertical black lines show the approximate end of the SMC period (at 150 days) and the approximate start of the 2013 transmission season (at 270 days).

Malaria incidence over the study period is shown in Figure 3. Kaplan‐Meier and Nelson‐Aalen plots of malaria incidence by randomisation group are shown in Figure 4. During the period when SMC was administered, there were 207 malaria episodes in 950.0 person‐years of follow‐up. 157 children experienced a single episode of malaria, 23 children experienced two episodes, and one child in the AL group experienced four episodes. Relative to the AL only group, the adjusted hazard ratio (aHR) was 0.62 (95% CI: 0.41, 0.93), *P* = 0.020 in the SMC group, a protective efficacy of 38.5% (95% CI 7.28%, 59.2%); the aHR was 1.18 (95% CI: 0.83, 1.67), *P* = 0.356 in the DP group (Table 4). Among children who received all five cycles of SMC or placebo, the aHR in the SMC group relative to the AL only group was 0.53 (95% CI: 0.29, 0.95), a protective efficacy of 47.4% (4.96%, 70.9%), *P* = 0.033. Although based on small numbers, the mean interval between recurrent episodes was greater in the DP group (*n* = 14 episodes, 64.1 days in the DP group, *vs. n* = 8 episodes, 34.8 days in the AL group and *n* = 4, 27.0 days in the SMC group, *P* = 0.017 for DP *vs*. AL, *P* = 0.63 for SMC *vs*. AL).

**Table 4:** Malaria incidence

	Malaria episodes	Person‐years	Incidence rate^a^	Hazard ratio (95% CI)	*P*‐value	Adjusted hazard ratio (95% CI)	*P*‐value
SMC period							
All children							
AL group	72	314.0	229.3	[reference]	–	[reference]	–
DP	90	318.9	282.2	1.23 (0.87, 1.73)	0.234	1.18 (0.83, 1.67)	0.356
SMC	45	317.2	141.9	0.62 (0.41, 0.92)	0.019	0.62 (0.41, 0.93)	0.020
Received five rounds							
AL only	41	137.6	298.0	[reference]	–	[reference]	–
DP	54	139.7	386.4	1.29 (0.82, 2.05)	0.274	1.16 (0.72, 1.87)	0.540
SMC	19	122.6	155.0	0.52 (0.29, 0.92)	0.026	0.53 (0.29, 0.95)	0.033
Post‐SMC period							
All children							
AL only	31	309.6	100.1	[reference]	–	[reference]	–
DP	52	305.2	170.4	1.70 (1.01, 2.86)	0.045	1.34 (0.86, 2.10)	0.199
SMC	52	301.4	172.5	1.73 (1.00, 2.99)	0.050	1.36 (0.86, 2.14)	0.187
Received five rounds							
AL only	23	152.6	150.7	[reference]	–	[reference]	–
DP	37	153.0	241.8	1.61 (0.87, 3.00)	0.131	1.22 (0.75, 1.98)	0.418
SMC	38	128.6	295.6	1.97 (1.04, 3.73)	0.038	1.44 (0.86, 2.42)	0.166
Whole study period							
All children							
AL only	103	623.6	165.2	[reference]	–	[reference]	–
DP	142	624.1	227.5	1.37 (1.00, 1.88)	0.047	1.23 (0.92, 1.65)	0.168
SMC	97	618.5	156.8	0.95 (0.67, 1.33)	0.755	0.88 (0.65, 1.20)	0.428
Received five rounds							
AL only	64	290.2	220.6	[reference]	–	[reference]	–
DP	91	292.8	310.8	1.41 (0.93, 2.14)	0.108	1.20 (0.83, 1.74)	0.337
SMC	57	251.1	227.0	1.03 (0.65, 1.62)	0.913	0.91 (0.62, 1.35)	0.644

a Rate per 1000 person‐years. SMC period: From date of receipt of first SMC or placebo course, until 1 month after the last SMC course. Post‐SMC period: from 1 month after last SMC course until the end of the study.

During the post‐SMC period, including the start of the following transmission season in 2013, there were 135 episodes of malaria in 916.2 person‐years of follow‐up. Relative to the AL only group, incidence of malaria appeared higher in the SMC group and the DP group: crude HRs 1.73 (95% CI: 1.00, 2.99), *P* = 0.050 and 1.70 (95% CI: 1.01, 2.86), *P* = 0.045, respectively. However, after adjusting for covariates (age, sex, SES, community and ITN use) aHRs were 1.36 (95% CI: 0.86, 2.14), *P* = 0.187 and 1.34 (95% CI: 0.86, 2.10), *P* = 0.199, respectively.

Over the whole study period, including the SMC period, the dry season and the first few months of the 2013 transmission season, 2143 children never experienced malaria, 198 children had one malaria episode, 40 children experienced two episodes, and 13, 5 and 1 children experienced 3, 4 and 5 episodes, respectively. Compared to the AL only group, the aHR was 0.88 (95% CI 0.65, 1.20), *P* = 0.428 in the SMC group and 1.23 (95% CI 0.92, 1.65), *P* = 0.168 in the DP group. There was no evidence of a difference in the interval between recurrent episodes between groups over the whole study period.

## Discussion

We investigated an extended programme of seasonal malaria chemoprevention, and case management with a long‐acting ACT in an area of Ghana with a long rainy season with two peaks. Strengths of this study are that a relatively large cohort of children was monitored, the interventions were successfully delivered, and it was possible to ensure a continuous supply of drugs and diagnostic tests to community health workers who documented malaria incidence. Limitations of this study are that not all children were followed over the entire study period, that randomisation did not achieve perfect balance in factors known to be associated with malaria, that for practical reasons it was necessary for the study to be open‐label with respect to the case‐management regimen and that coverage of the full complement of SMC cycles was relatively low.

Malaria incidence was lower than expected: 196.4 per 1000 child‐years over the study period in children who received placebo SMC, compared to 700 per 1000 person‐years assumed in the sample size calculation. Several factors may have contributed to this. Firstly, due to a recent distribution campaign, around 80% of children were regularly sleeping under an insecticide‐treated net. Secondly, increased membership of Ghana's National Health Insurance scheme (allowing free treatment at health centres) may have contributed to lower patronage of CHWs, as in a recent study in Burkina Faso 20. Thirdly, the practice of CHWs referring children with a negative RDT result to health facilities may have proved less popular with parents/caregivers than presumptive treatment, as seen elsewhere 21. Staff were stationed at health centres to document episodes that presented directly, but there may also have been more use of the private sector when diagnostics were introduced 22. Due to the lower incidence of malaria, this study was not adequately powered to evaluate robustly the benefit of the longer period of post‐treatment prophylaxis provided by DP. During the SMC period, the interval between episodes was longer in the DP group, but few children experienced more than one episode, and this difference was not seen over the whole study period.

The protective efficacy of SMC was lower than expected during the SMC period, at around 38%, with no significant protection over the whole study period, although this is partly diluted by the onset of the 2013 transmission season a few months before the study ended. The lower efficacy may partly be due to relatively low coverage of all five SMC cycles (36.4% in the SMC group), but even among children who received five cycles of SMC, protective efficacy was around 47% during the SMC period, lower than the efficacy of 70‐80% seen in other studies of monthly SP‐AQ 4, 6, 7, or monthly artesunate‐amodiaquine 10. This could be a chance finding, because malaria incidence was low and confidence intervals for the efficacy are wide. However, if correct, it could suggest failure to complete the 3‐day course of SMC (SP plus AQ on day one, AQ only on days two and three): although SP alone retains reasonably high efficacy in most of West Africa, the efficacy of SP plus AQ is higher 5. Adherence data were collected but implausibly high values of adherence were reported, as seen elsewhere 23. Overall efficacy might also be lower because incidence is relatively low in the study area as a whole: 7 of the 13 study communities have an annual incidence of malaria below 100 cases per 1000 child‐years at risk, the lower limit suggested for SMC to be cost‐effective 8. In three small communities with a high prevalence of malaria at baseline and high incidence during the transmission season (Korase, Sarpeh and Timeabu) the protective efficacy of SMC (any number of cycles) was more consistent with earlier studies: 69.7% (95% CI: 22.7%, 88.1%). Potentially supporting this is the fact that there was no benefit of SMC on anaemia, in contrast to findings from SMC studies in high transmission areas 6, 7 but agreeing with areas of lower transmission 1.

Low efficacy may also be a consequence of the timing of administration of the first cycle of SMC. At the baseline survey in July, prevalence was around 26%, suggesting that the transmission season was well underway by this point. Although SP‐AQ is likely to have very good curative efficacy in West Africa 24, 25, the benefit of SMC will likely be greater where children are protected prior to exposure each year. If SMC was started too late in 2012 relative to the start of the transmission season, this suggests that five cycles of SMC will be insufficient in this setting, as there was also some malaria incidence in January 2013. Seven or possibly eight cycles could cover the period between May and January, but this raises new questions of cost‐effectiveness, and the safety and acceptability of a substantial additional number of SMC cycles.

Operationally, delivery of a larger number of SMC cycles is more challenging, as the additional drugs needed bring associated issues of cost, supply and storage. There may also be more potential for campaign fatigue where drugs are administered over a longer period, both on the part of parents/caregivers and their children, and on the part of delivery teams. This appeared to be the case here, even in the context of a research study, with decreasing coverage at the second and third cycles. However, the increased coverage at the fourth and fifth cycles, achieved by following up children at home, agrees with previous results showing that door‐to‐door delivery by community‐based health workers can achieve high SMC coverage 26, 27 and underlines the need for close supervision of SMC where it is delivered.

## Conclusion

Five cycles of SMC administered during the peak of the malaria transmission season reduced the burden of malaria by around 50% during the rainy season. Although this is lower than might be expected with monthly SMC, and although the burden of malaria has fallen compared to earlier studies undertaken in this area, this still represents an important public health impact. Lower efficacy may be partly attributable to use of other protective measures and lower SMC coverage. It is also likely that five monthly cycles of SMC were insufficient to adequately address the full malaria transmission season and that an additional number of cycles would be needed to achieve higher levels of protection using SMC in this and similar areas. Further research is needed to consider the role of SMC and other forms of chemoprevention in areas with seasonal malaria that extends over 6 or more months of the year.

## Supporting information


**Figure S1** Prevalence of malaria at baseline by study community
**Figure S2**. Distribution of haemoglobin by study group in July 2012, January 2013 and July 2013
**Table S1**. Distribution of children by community and study group.
**Table S2**. Malaria incidence during the SMC period according to the number of rounds of SMC/placebo received.
**Table S3**. Malaria incidence during the remainder of the post‐SMC period according to the number of rounds of SMC/placebo received
**Table S4**. Malaria incidence over the whole study period according to the number of rounds of SMC/placebo received
**Table S5**. Analysis of malaria incidence during the malaria transmission season: results from random effects Poisson regression
**Table S6**. Symptoms at January survey
**Table S7**. Symptoms at July Survey
