# Supplementary material for: Seasonal malaria chemoprevention in an area of extended seasonal transmission in Ashanti, Ghana: an individually randomised clinical trial
Source: Trop Med Int Health. 2015 Dec 16;21(2):224–35. doi: 10.1111/tmi.12642 (PMC4982104; doi:10.1111/tmi.12642)
Supplement: Supplementary file 1 — Figure S1 Prevalence of malaria at baseline by study community Figure S2. Distribution of haemoglobin by study group in July 2012, January 2013 and July 2013 Table S1. Distribution of children by community and study group. Table S2. Malaria incidence during the SMC period according to the number of rounds of SMC/placebo received. Table S3. Malaria incidence during the remainder of the post‐SMC period according to the number of rounds of SMC/placebo received Table S4. Malaria incidence over the whole study period according to the number of rounds of SMC/placebo received Table S5. Analysis of malaria incidence during the malaria transmission season: results from random effects Poisson regression Table S6. Symptoms at January survey Table S7. Symptoms at July Survey [file TMI-21-224-s001.docx]

Supplementary Tables and Figures

Supplementary tables

Table S1. Distribution of children by community and study group.

Table S2. Malaria incidence during the SMC period according to the number of rounds of SMC/placebo received.

Table S3. Malaria incidence during the remainder of the post-SMC period according to the number of rounds of SMC/placebo received

Table S4. Malaria incidence over the whole study period according to the number of rounds of SMC/placebo received

Table S5. Analysis of malaria incidence during the malaria transmission season: results from random effects Poisson regression

Table S6. Symptoms at January survey

Table S7. Symptoms at July Survey

Supplementary Figures

Figure S1: Prevalence of malaria at baseline by study community

Figure S2. Distribution of haemoglobin by study group in July 2012, January 2013 and July 2013

Table S1. Distribution of children by community and study group.

|  | AL group | | DP group | | SMC group | |
| --- | --- | --- | --- | --- | --- | --- |
| Community | N | % | N | % | N | % |
| Abenase | 52 | 6.5 | 54 | 6.8 | 54 | 6.8 |
| Achinakrom | 84 | 10.5 | 80 | 10 | 87 | 10.9 |
| Deduako | 62 | 7.8 | 62 | 7.8 | 59 | 7.4 |
| Donaso | 26 | 3.3 | 27 | 3.4 | 26 | 3.3 |
| Donyina | 81 | 10.1 | 82 | 10.3 | 82 | 10.3 |
| Edwinase | 42 | 5.3 | 38 | 4.8 | 36 | 4.5 |
| Essienimpong | 119 | 14.9 | 118 | 14.8 | 121 | 15.1 |
| Korase | 15 | 1.9 | 21 | 2.6 | 20 | 2.5 |
| Kwaso | 133 | 16.6 | 130 | 16.3 | 134 | 16.8 |
| Odaho | 39 | 4.9 | 39 | 4.9 | 37 | 4.6 |
| Onwe | 109 | 13.6 | 106 | 13.3 | 107 | 13.4 |
| Sarpeh | 28 | 3.5 | 31 | 3.9 | 26 | 3.3 |
| Timeabu | 10 | 1.3 | 12 | 1.5 | 11 | 1.4 |
| Total | 800 |  | 800 |  | 800 |  |

Figure S1: Prevalence of malaria at baseline by study community

Prevalence of malaria in July 2012. The size of the under five population in each study community according to the census is indicated in brackets.

Figure S2. Distribution of haemoglobin by group in A) July 2012, B) January 2013 and C) July 2013

Table S2. Malaria incidence during the SMC period according to the number of rounds of SMC/placebo received.

| **SMC period** | | **Malaria episodes** | **Person-years** | **Incidence Rate^*^** | **Hazard Ratio**  **(95% CI)** | **P-value** | **Adjusted Hazard Ratio**  **(95% CI)** | **P-value** |
| --- | --- | --- | --- | --- | --- | --- | --- | --- |
| All children | AL group | 72 | 314.0 | 229.3 | [reference] | - | [reference] | - |
|  | DP | 90 | 318.9 | 282.2 | 1.23 (0.87, 1.73) | 0.234 | 1.18 (0.83, 1.67) | 0.356 |
|  | SMC | 45 | 317.2 | 141.9 | 0.62 (0.41, 0.92) | 0.019 | 0.62 (0.41, 0.93) | 0.020 |
|  |  |  |  |  |  |  |  |  |
| At least 2 | AL only | 72 | 303.3 | 237.4 | [reference] | - | [reference] | - |
| rounds | DP | 89 | 309.0 | 288.1 | 1.21 (0.86, 1.70) | 0.267 | 1.16 (0.81, 1.64) | 0.417 |
|  | SMC | 42 | 308.9 | 136.0 | 0.57 (0.38, 0.86) | 0.008 | 0.59 (0.39, 0.89) | 0.013 |
|  |  |  |  |  |  |  |  |  |
| At least 3 | AL only | 64 | 270.1 | 237.0 | [reference] | - | [reference] | - |
| rounds | DP | 83 | 280.3 | 296.1 | 1.25 (0.87, 1.79) | 0.229 | 1.17 (0.81, 1.69) | 0.413 |
|  | SMC | 40 | 277.1 | 144.4 | 0.61 (0.40, 0.93) | 0.023 | 0.61 (0.39, 0.95) | 0.027 |
|  |  |  |  |  |  |  |  |  |
| At least 4 | AL only | 57 | 216.1 | 263.7 | [reference] | - | [reference] | - |
| rounds | DP | 79 | 228.0 | 346.5 | 1.31 (0.90, 1.92) | 0.161 | 1.23 (0.83, 1.82) | 0.297 |
|  | SMC | 35 | 217.4 | 161.0 | 0.61 (0.39, 0.96) | 0.033 | 0.61 (0.39, 0.97) | 0.038 |
|  |  |  |  |  |  |  |  |  |
| All 5 rounds | AL only | 41 | 137.6 | 298.0 | [reference] | - | [reference] | - |
| rounds | DP | 54 | 139.7 | 386.4 | 1.29 (0.82, 2.05) | 0.274 | 1.16 (0.72, 1.87) | 0.540 |
|  | SMC | 19 | 122.6 | 155.0 | 0.52 (0.29, 0.92) | 0.026 | 0.53 (0.29, 0.95) | 0.033 |

Table S3. Malaria incidence during the remainder of the post-SMC period according to the number of rounds of SMC/placebo received

| **Post-SMC period** | | **Malaria episodes** | **Person-years** | **Incidence Rate^*^** | **Hazard Ratio**  **(95% CI)** | **P-value** | **Adjusted Hazard Ratio**  **(95% CI)** | **P-value** |
| --- | --- | --- | --- | --- | --- | --- | --- | --- |
| All children | AL only | 31 | 309.6 | 100.1 | [reference] | - | [reference] | - |
|  | DP | 52 | 305.2 | 170.4 | 1.70 (1.01, 2.86) | 0.045 | 1.34 (0.86, 2.10) | 0.199 |
|  | SMC | 52 | 301.4 | 172.5 | 1.73 (1.00, 2.99) | 0.050 | 1.36 (0.86, 2.14) | 0.187 |
|  |  |  |  |  |  |  |  |  |
| At least 2 | AL only | 31 | 298.9 | 103.7 | [reference] | - | [reference] | - |
| rounds | DP | 52 | 297.6 | 174.7 | 1.68 (1.00, 2.83) | 0.049 | 1.33 (0.85, 2.08) | 0.214 |
|  | SMC | 50 | 294.5 | 169.8 | 1.64 (0.95, 2.85) | 0.078 | 1.30 (0.82, 2.05) | 0.259 |
|  |  |  |  |  |  |  |  |  |
| At least 3 | AL only | 30 | 268.5 | 111.7 | [reference] | - | [reference] | - |
| rounds | DP | 51 | 279.6 | 182.4 | 1.63 (0.96, 2.77) | 0.069 | 1.34 (0.85, 2.12) | 0.204 |
|  | SMC | 48 | 268.3 | 178.9 | 1.61 (0.91, 2.82) | 0.099 | 1.34 (0.84, 2.13) | 0.220 |
|  |  |  |  |  |  |  |  |  |
| At least 4 | AL only | 29 | 222.6 | 130.3 | [reference] | - | [reference] | - |
| rounds | DP | 48 | 238.1 | 201.6 | 1.55 (0.90, 2.66) | 0.113 | 1.26 (0.80, 1.98) | 0.317 |
|  | SMC | 46 | 215.9 | 213.0 | 1.64 (0.92, 2.92) | 0.094 | 1.31 (0.82, 2.09) | 0.253 |
|  |  |  |  |  |  |  |  |  |
| All 5 rounds | AL only | 23 | 152.6 | 150.7 | [reference] | - | [reference] | - |
| rounds | DP | 37 | 153.0 | 241.8 | 1.61 (0.87, 3.00) | 0.131 | 1.22 (0.75, 1.98) | 0.418 |
|  | SMC | 38 | 128.6 | 295.6 | 1.97 (1.04, 3.73) | 0.038 | 1.44 (0.86, 2.42) | 0.166 |

Table S4. Malaria incidence over the whole study period according to the number of rounds of SMC/placebo received

| **Whole Study period** | | **Malaria episodes** | **Person-years** | **Incidence Rate^*^** | **Hazard Ratio**  **(95% CI)** | **P-value** | **Adjusted Hazard Ratio**  **(95% CI)** | **P-value** |
| --- | --- | --- | --- | --- | --- | --- | --- | --- |
| All children | AL only | 103 | 623.6 | 165.2 | [reference] | - | [reference] | - |
|  | DP | 142 | 624.1 | 227.5 | 1.37 (1.00, 1.88) | 0.047 | 1.23 (0.92, 1.65) | 0.168 |
|  | SMC | 97 | 618.5 | 156.8 | 0.95 (0.67, 1.33) | 0.755 | 0.88 (0.65, 1.20) | 0.428 |
|  |  |  |  |  |  |  |  |  |
| At least 2 | AL only | 103 | 602.3 | 171.0 | [reference] | - | [reference] | - |
| rounds | DP | 141 | 606.6 | 232.5 | 1.36 (0.99, 1.86) | 0.057 | 1.21 (0.90, 1.62) | 0.206 |
|  | SMC | 92 | 603.5 | 152.5 | 0.89 (0.63, 1.26) | 0.511 | 0.84 (0.62, 1.15) | 0.276 |
|  |  |  |  |  |  |  |  |  |
| At least 3 | AL only | 94 | 538.6 | 174.5 | [reference] | - | [reference] | - |
| rounds | DP | 134 | 560.0 | 239.3 | 1.37 (0.99, 1.91) | 0.061 | 1.22 (0.90, 1.66) | 0.202 |
|  | SMC | 88 | 545.4 | 161.4 | 0.92 (0.64, 1.33) | 0.665 | 0.87 (0.63, 1.20) | 0.397 |
|  |  |  |  |  |  |  |  |  |
| At least 4 | AL only | 86 | 438.7 | 196.0 | [reference] | - | [reference] | - |
| rounds | DP | 127 | 466.1 | 272.5 | 1.39 (0.99, 1.96) | 0.060 | 1.25 (0.91, 1.72) | 0.170 |
|  | SMC | 81 | 433.3 | 187.0 | 0.95 (0.65, 1.40) | 0.797 | 0.88 (0.63, 1.24) | 0.471 |
|  |  |  |  |  |  |  |  |  |
| All 5 rounds | AL only | 64 | 290.2 | 220.6 | [reference] | - | [reference] | - |
| rounds | DP | 91 | 292.8 | 310.8 | 1.41 (0.93, 2.14) | 0.108 | 1.20 (0.83, 1.74) | 0.337 |
|  | SMC | 57 | 251.1 | 227.0 | 1.03 (0.65, 1.62) | 0.913 | 0.91 (0.62, 1.35) | 0.644 |

Table S5. Analysis of malaria incidence during the malaria transmission season: results from random effects Poisson regression

| **Random Effects Poisson model** | **Group** | **IRR (95% CI)** | **P-value** |
| --- | --- | --- | --- |
| **All children** |  |  |  |
| Crude | AL group | [reference] |  |
|  | DP group | 1.31 (0.88, 1.94) | 0.182 |
|  | SMC group | 0.58 (0.37, 0.91) | 0.017 |
|  |  |  |  |
| Adjusted for month | AL group | [reference] |  |
|  | DP group | 1.29 (0.88, 1.88) | 0.194 |
|  | SMC group | 0.59 (0.39, 0.91) | 0.018 |
|  |  |  |  |
| Adjusted for month, age group, sex, community, SES, ITN | AL group | [reference] |  |
|  | DP group | 1.25 (0.85, 1.84) | 0.262 |
|  | SMC group | 0.60 (0.38, 0.93) | 0.022 |
|  |  |  |  |
| **Children who received 5 rounds of SMC** |  |  |  |
| Crude | AL group | [reference] |  |
|  | DP group | 1.38 (0.80, 2.38) | 0.240 |
|  | SMC group | 0.47 (0.25, 0.91) | 0.026 |
|  |  |  |  |
| Adjusted for month | AL group | [reference] |  |
|  | DP group | 1.37 (0.81, 2.31) | 0.236 |
|  | SMC group | 0.49 (0.26, 0.93) | 0.028 |
|  |  |  |  |
| Adjusted for month, age group, sex, community, SES, ITN | AL group | [reference] |  |
|  | DP group | 1.21 (0.71, 2.07) | 0.485 |
|  | SMC group | 0.49 (0.26, 0.93) | 0.030 |

In all models, there was strong evidence against the null that alpha, the overdispersion parameter = 0 (P<0.001 in all cases).

**Table S6. Symptoms at January survey**

| Symptom | AL group | | DP group | | SMC group | | P-value |
| --- | --- | --- | --- | --- | --- | --- | --- |
|  | N | % | N | % | N | % |  |
| N | 592 |  | 594 |  | 588 |  |  |
|  |  |  |  |  |  |  |  |
| Febrile* | 18 | 3.3 | 11 | 2.0 | 13 | 2.4 | 0.368 |
| History of fever | 39 | 6.6 | 41 | 6.9 | 39 | 6.6 | 0.975 |
| Malaria infection | 120 | 20.3 | 116 | 19.5 | 73 | 12.4 | <0.001 |
| Fever and malaria | 13 | 2.2 | 19 | 3.2 | 16 | 2.7 | 0.603 |
|  |  |  |  |  |  |  |  |
| **Reported symptoms** |  |  |  |  |  |  |  |
| Sick or unwell | 58 | 9.8 | 60 | 10.1 | 70 | 11.9 | 0.454 |
| Fever | 40 | 6.8 | 37 | 6.2 | 52 | 8.8 | 0.190 |
| Vomiting | 13 | 2.2 | 10 | 1.7 | 10 | 1.7 | 0.754 |
| Diarrhoea | 14 | 2.4 | 10 | 1.7 | 16 | 2.7 | 0.473 |
| Refusal of feeds | 10 | 1.7 | 8 | 1.3 | 5 | 0.9 | 0.437 |
| Chills | 3 | 0.5 | 7 | 1.2 | 4 | 0.7 | 0.400 |
| Headache | 11 | 1.9 | 16 | 2.7 | 20 | 3.4 | 0.259 |
| Abdominal pain | 2 | 0.3 | 3 | 0.5 | 2 | 0.3 | 0.872 |
| General weakness | 1 | 0.2 | 4 | 0.7 | 1 | 0.2 | 0.227 |
| Cough | 26 | 4.4 | 35 | 5.9 | 25 | 4.3 | 0.349 |
| Runny nose | 22 | 3.7 | 32 | 5.4 | 32 | 5.4 | 0.298 |
| Breathing problems | 2 | 0.3 | 1 | 0.2 | 0 | 0 | 0.367 |
| Pallor | 3 | 0.5 | 0 | 0 | 0 | 0 | 0.049 |
| Convulsion | 1 | 0.2 | 0 | 0 | 0 | 0 | 0.367 |
| Jaundice | 1 | 0.2 | 1 | 0.2 | 0 | 0 | 0.608 |
| Dark urine | 1 | 0.2 | 0 | 0 | 0 | 0 | 0.367 |

Symptom data were not available from 2 respondents in the AL group. * Temperature ≥37.5° C Measured temperature was missing from 145 children (53 in the AL group, 48 in the DP group and 44 in the SMC group). 18 children were missing data for measured temperature and history of fever, or malaria parasitaemia (13 in the AL group, 1 in the DP group and 4 in the SMC group).

**Table S7. Symptoms at July Survey**

| Symptom | AL group | | DP group | | SMC group | | P-value |
| --- | --- | --- | --- | --- | --- | --- | --- |
|  | N | % | N | % | N | % |  |
| N | 530 |  | 518 |  | 505 |  |  |
|  |  |  |  |  |  |  |  |
| Febrile* | 26 | 4.9 | 27 | 5.4 | 18 | 3.5 | 0.338 |
| History of fever | 103 | 19.4 | 87 | 16.8 | 103 | 20.4 | 0.305 |
| Malaria infection | 116 | 22 | 106 | 20.5 | 116 | 23.2 | 0.580 |
| Fever and malaria | 34 | 6.5 | 32 | 6.2 | 43 | 8.6 | 0.259 |
|  |  |  |  |  |  |  |  |
| **Reported symptoms** |  |  |  |  |  |  |  |
| Sick or unwell | 172 | 32.5 | 152 | 29.3 | 152 | 30.2 | 0.527 |
| Fever | 109 | 20.6 | 95 | 18.3 | 98 | 19.4 | 0.661 |
| Vomiting | 35 | 6.6 | 20 | 3.9 | 29 | 5.8 | 0.134 |
| Diarrhoea | 62 | 11.7 | 49 | 9.5 | 49 | 9.7 | 0.428 |
| Refusal of feeds | 48 | 9.1 | 33 | 6.4 | 40 | 7.9 | 0.266 |
| Chills | 27 | 5.1 | 12 | 2.3 | 26 | 5.2 | 0.034 |
| Headache | 122 | 23 | 111 | 21.4 | 112 | 22.2 | 0.826 |
| Abdominal pain | 31 | 5.8 | 25 | 4.8 | 26 | 5.2 | 0.752 |
| General weakness | 20 | 3.8 | 13 | 2.5 | 18 | 3.6 | 0.470 |
| Cough | 136 | 25.7 | 118 | 22.8 | 119 | 23.6 | 0.532 |
| Runny nose | 154 | 29.1 | 118 | 22.8 | 129 | 25.6 | 0.067 |
| Breathing problems | 26 | 4.9 | 21 | 4.1 | 19 | 3.8 | 0.640 |
| Pallor | 2 | 0.4 | 0 | 0 | 0 | 0 | 0.145 |
| Convulsion | 0 | 0 | 0 | 0 | 0 | 0 | - |
| Jaundice | 0 | 0 | 0 | 0 | 0 | 0 | - |
| Dark urine | 3 | 0.6 | 2 | 0.4 | 3 | 0.6 | 0.879 |

2 individuals were missing symptoms at the July survey (one in the DP group and one in the SMC group). . * Temperature ≥37.5° C. 10 individuals were missing measured temperature (1 in the AL group, 7 in the DP group, and 2 in the SMC group). 10 individuals were missing malaria status (3 in the AL group, 2 in the DP group and 5 in the SMC group).
